# Characterization of organelle DNA degradation mediated by DPD1 exonuclease in the rice genome-edited line

**DOI:** 10.1007/s11103-024-01452-x

**Published:** 2024-06-10

**Authors:** Md. Faridul Islam, Hiroshi Yamatani, Tsuneaki Takami, Makoto Kusaba, Wataru Sakamoto

**Affiliations:** 1 Institute of Plant Science and Resources, Okayama University, 2-20-1 Chuo, Kurashiki, Okayama 710-0046 Japan; 2 Department of Quantum-Applied Biosciences, Takasaki Institute for Advanced Quantum Science, Foundational Quantum Technology Research Directorate, National Institutes for Quantum Science and Technology (QST), 1233, Watanuki, Takasaki, Gunma 370-1292 Japan; 3 Graduate School of Integrated Sciences for Life, Hiroshima University, 1-4-3 Kagamiyama, Higashi-Hiroshima, Hiroshima, 739-8528 Japan

**Keywords:** Exonuclease, Leaf senescence, Mitochondria, Organelle DNA degradation, Plastids, Pollen, Rice (*Oryza sativa*)

## Abstract

Mitochondria and plastids, originated as ancestral endosymbiotic bacteria, contain their own DNA sequences. These organelle DNAs (orgDNAs) are, despite the limited genetic information they contain, an indispensable part of the genetic systems but exist as multiple copies, making up a substantial amount of total cellular DNA. Given this abundance, orgDNA is known to undergo tissue-specific degradation in plants. Previous studies have shown that the exonuclease DPD1, conserved among seed plants, degrades orgDNAs during pollen maturation and leaf senescence in *Arabidopsis*. However, tissue-specific orgDNA degradation was shown to differ among species. To extend our knowledge, we characterized DPD1 in rice in this study. We created a genome-edited (GE) mutant in which *OsDPD1* and *OsDPD1-like* were inactivated. Characterization of this GE plant demonstrated that DPD1 was involved in pollen orgDNA degradation, whereas it had no significant effect on orgDNA degradation during leaf senescence. Comparison of transcriptomes from wild-type and GE plants with different phosphate supply levels indicated that orgDNA had little impact on the phosphate starvation response, but instead had a global impact in plant growth. In fact, the GE plant showed lower fitness with reduced grain filling rate and grain weight in natural light conditions. Taken together, the presented data reinforce the important physiological roles of orgDNA degradation mediated by DPD1.

**Supplementary Information:**

The online version contains supplementary material available at 10.1007/s11103-024-01452-x.

## Key message

Organelle exonuclease DPD1 operates plastid and mitochondrial DNA degradation during pollen development in rice as well as *Arabidopsis*, but its action in senescing leaves may differ between the species.

**Supplementary Information:**

The online version contains supplementary material available at 10.1007/s11103-024-01452-x.

## Introduction

More than a billion years ago, mitochondria and chloroplasts (plastids) originated from endosymbiotic events involving an ancestral α-proteobacterium for mitochondria and a cyanobacterium for chloroplasts (Dyall et al. 2004). Both organelles retain their genomes and perform processes such as DNA replication, transcription, and translation leading to protein synthesis within each organelle, even though the majority of genes found in the primitive endosymbionts have undergone relocation to the nuclear genome of plants (Gray 1999; Kleine et al. 2009). The current mitochondrial DNA (mtDNA) and plastid DNA (ptDNA) genomes encode only a limited number of genes in most angiosperms; for example, only 87 protein-coding genes are encoded in the *Arabidopsis*154 kb plastid genome (Sato et al. 1999). A unique and intriguing characteristic of organelle DNA (orgDNA) is that it exists as multiple copies and undergoes tissue-specific degradation (Kuroiwa 2010; Oldenburg and Bendich 2015; Sakamoto and Takami 2018). Observations related to orgDNA degradation derive mostly from cytological studies (e.g. Nagata 2010 and references therein), whereas recent findings have shed light on the nucleases that actively degrade ptDNAs and mtDNAs (Ma et al. 2018; Matsushima et al. 2011; Sakamoto and Takami 2018, 2023).

There are two important aspects to be considered for orgDNA degradation, particularly for ptDNAs. One is the uniparental inheritance of ptDNAs, in contrast to the biparental inheritance of chromosomal DNAs. The majority of angiosperm species (about 80%) show maternal inheritance (Corriveau and Coleman 1988; Zhang et al. 2003), and accumulating evidence demonstrates that several critical steps exist to mutually control maternal inheritance in the development of male germ lines (pollen) (Nagata 2010; Sakamoto and Takami 2023 for review). Maternal inheritance is predominantly determined by the exclusion of plastids themselves and/or the degradation of ptDNAs in pollen. In particular, a recent report in tobacco revealed that the degradation of ptDNAs, mediated by the organelle exonuclease DPD1, plays a role in ensuring maternal inheritance (Chung et al. 2023). DPD1 was originally identified as a Mg^2+^-dependent 3’-to-5’exo-deoxyribonuclease through a genetic screen for the mutants showing orgDNA retention in *Arabidopsis* pollen grains. Indeed, DPD1 was shown to be dual targeted to mitochondria and plastids, and it degrades orgDNAs during pollen maturation (Matsushima et al. 2011). It is conserved not only in angiosperms but also gymnosperms, whereas it is absent in mosses and green algae. Given its prokaryotic nature as homologous to a proof-reading subunit (DnaQ) of bacterial DNA polymerase I, DPD1 may have emerged during the evolution of streptophytes to secure maternal inheritance. DPD1 is distinct because most of the nucleases related to plant cell death previously identified in plants are predominantly staphylococcal-like or S1-like type endonucleases (Sakamoto and Takami 2014).

In addition to maternal inheritance, another important aspect of ptDNA is its abundance in vegetative tissues. Despite its small genome and limited genetic capacity, ptDNAs account for a substantial proportion of total DNA within cells, because ptDNA exists in multiple copies as chloroplast DNA (cpDNA) (Sakamoto and Takami 2018, 2023).The percentage of ptDNA in total cellular DNA changes during development and varies from tissue to tissue. As such, in leaf tissues ptDNAs can make up more than 20% of the total DNA(Lamppa and Bendich 1979; Rauwolf et al. 2010; The Arabidopsis Genome Initiative 2000).Chloroplasts constitute a major source of cellular phosphorus in leaves, because of the large amount of nucleic acids (Dean & Leech 1982; Veneklaas et al. 2012). Nucleic acids and phospholipids comprise the majority of phosphate esters in leaves (Smith et al. 2015; Veneklaas et al. 2012). The cpDNA pool may function as an internal phosphate reservoir in the vegetative tissues (Sears and VanWinkle-Swift 1994; Yehudai-Resheff et al. 2007), if it is degraded into smaller molecules that can be redistributed to other upper tissues. Our previous work elucidated such a phosphate reservoir function of ptDNAs, by studying *Arabidopsis dpd1* mutants. We demonstrated that DPD1 is upregulated during leaf senescence, involved in the efficient use of phosphate, and affects better seed setting in phosphate-depleted conditions (Takami et al. 2018).

The discovery of DPD1 and tissue-specific degradation underscores its genetic and physiological impact in both vegetative and reproductive growth. However, the precise role of orgDNA degradation might vary between species, as evidenced by differing modes of orgDNA inheritance. Moreover, a recent study revealed orgDNA degradation related to biotic stress, showing that a pathogenic factor originating from *Begomovirus* upregulated the expression of *DPD1*, resulting in vein-clearing, mosaic, and chlorotic symptoms in tobacco plants (Nair et al. 2023). Here, to characterize *DPD1* in more detail, we attempted to generate a mutant lacking DPD1 function in rice by genome editing. Rice (*Oryza sativa* L.) is a highly important global cereal crop, which contributes approximately 25% of the global dietary energy supply for human consumption (Kusano et al. 2015; Miura et al. 2010). However, there is limited understanding regarding the orgDNA degradation mechanism in rice. In this study, we focused on *DPD1* genes in rice and their participation in orgDNA degradation. Characterization of the response to phosphate starvation indicated that the influence of DPD1 nuclease in vegetative tissues is different between rice and *Arabidopsis*. As for pollen, orgDNA was shown to be degraded by DPD1 during pollen maturation in rice. Conserved roles of orgDNA degradation across species are discussed.

## Methods

### Plant materials and growth conditions

In this study, Nipponbare (NB) was used as the wild-type rice variety (*Oryza sativa* L.), and a genome-edited (GE) line with *DPD1* knockouts was generated from NB. For general growth, rice seeds without husks were germinated in a petri-dish with wet filter papers, placed in an incubator set at 27–30°C and 10-h light period (100–125 µmol/m^2^/s), and subsequently transplanted into pots or a small chamber for hydroponic culture. Pot-grown T3 or T4 plants were cultivated (Ube granular soil No. 2) in a closed greenhouse at the Institute of Plant Science and Resources (Kurashiki City, Okayama, Japan, latitude: 34 °35’31’’ N, longitude: 133 °46’7’’ E) in which temperature was controlled at 27–30 °C. Sunlight was the sole light source when plants were grown in the summer (from May to October), whereas light was supplemented using LED lamps in the winter at night to prevent early heading. Seeds were harvested from each GE individual, dried at 30 °C for 30 days, and stored at 15 °C until use. Pot-grown plants were also cultivated (Ube granular soil No. 1) in an greenhouse at the Graduate School of Integrated Sciences for Life, Hiroshima University (Higashi-Hiroshima City, latitude: 34 °24’07’’ N, longitude: 132 °43’01 E) in the summer of 2022 and 2023 with natural sunlight. Plants harvested in greenhouses were dried for more than 3weeks and subjected to measurements for agronomical traits. Data were collected from 16 plants at Kurashiki and 10 plants at Hiroshima.

For hydroponic culture of rice seedlings, we followed the protocol described previously (Kuroda and Ikenaga 2015). Germinated seeds were placed on the top surface of a small hand-made plastic chamber (11 cm ×14 cm width ×14.5 cm height) filled with 1.5 L of 1/10 Murashige and Skoog (MS) Medium. Where necessary, Pi concentration was modified for phosphate depleted (-P) or phosphate enriched (+ P) conditions by supplementing with KH_2_PO_4_ at 1.25 mM. The medium for hydroponic culture was replaced twice a week. For dark-induced leaf senescence, detached flag leaves of pot-grown plants were placed in sterilized water in a titer plate and incubated in darkness. Chlorophyll content in leaves was measured using a SPAD-502 plus instrument (Konica Minolta) at different timepoints (0, 1, 2, 3, 4, 5, 6, 7, and 8 weeks) after heading.

### Generation of the genome-edited mutant line

**Fig. 1:**
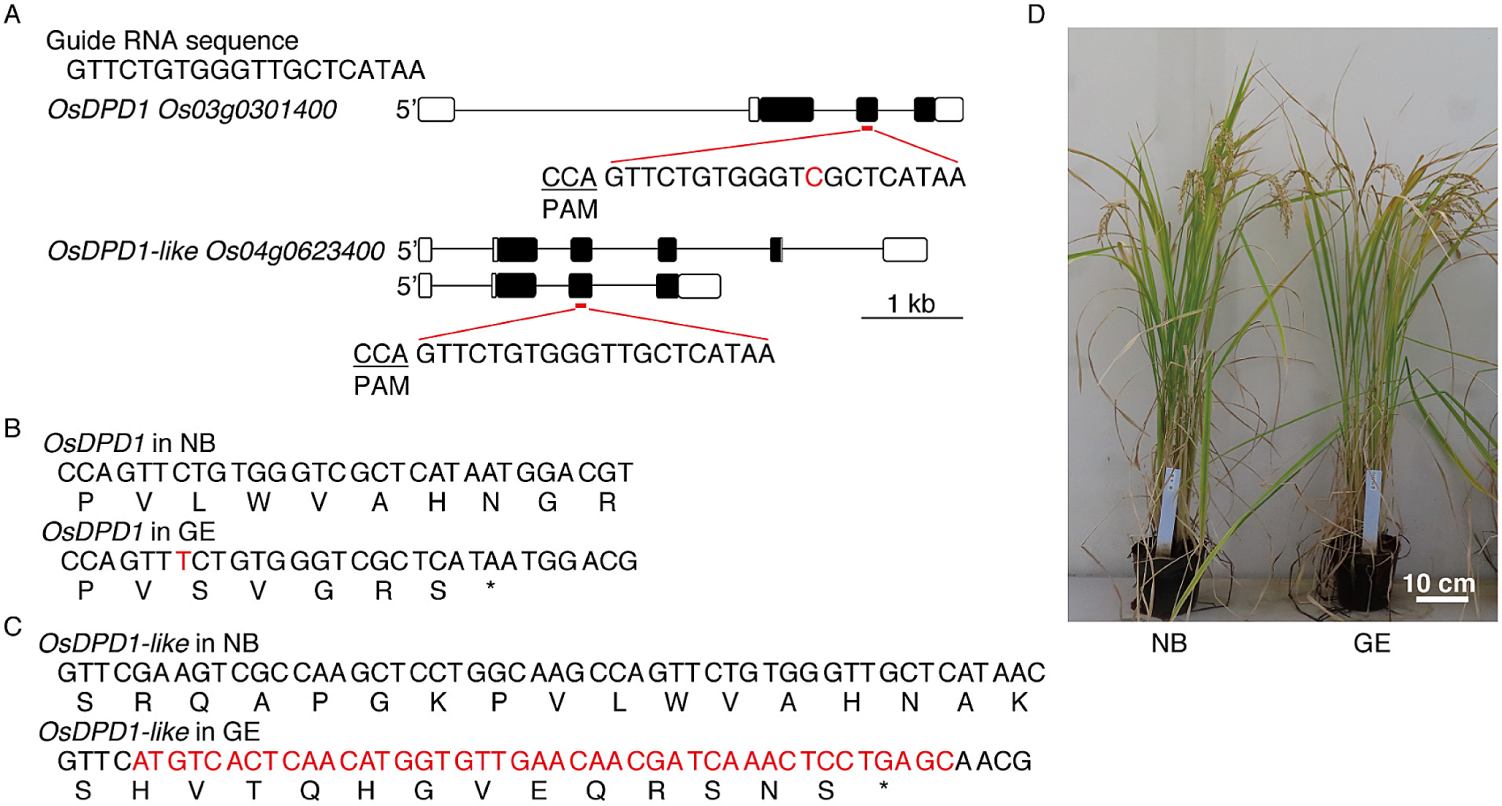
Schematic diagram showing the gene models of *OsDPD1*and *OsDPD1-like* and the GE line generated in this study. (**A**) Gene models of *OsDPD1* and *OsDPD1-like* are shown. Closed boxes indicate coding regions, and open boxes indicate untranslated regions. The target of the 20-bp gRNA sequence employed in this study is highlighted in red, and the zoomed-in view shows the corresponding sequences (note there is one-base mismatch in *OsDPD1*). (**B**) Nucleotide arrangement around the target sequence of *OsDPD1*. One T insertion in GE is indicated by red, and the resulting amino-acid change is shown below the nucleotide. (**C**) Nucleotide arrangement around the target sequence of *OsDPD1-like*. Rearrangements in GE are indicated in red, and the resulting amino-acid change is shown below the nucleotides. (**D**) Photographs of NB and GE plants grown in a greenhouse

A guide RNA was designed to target both *OsDPD1* (Os03g0301400) and *OsDPD1-like* (Os04g0623400) genes in a relatively conserved region where *OsDPD1-like* and *OsDPD1* show a perfect match and a single base mismatch, respectively (Fig. 1). DNA oligomers for the guide RNA, prepared from a pair of primers (Os04g0623400 CRISPR F1: 5’-GTTGTTATGAGCAACCCACAGAAC-3’, Os04g0623400 CRISPR R1: 5’-AAACGTTCTGTGGGTTGCTCATAA-3’, the linker sequences are shown as underlined), were cloned into the *Bbs*I sites of a pU6gRNA vector (Mikami et al. 2015). The *Asc*I-*Pac*I fragment of the resultant pU6gRNA construct was cloned into the *Asc*I-*Pac*I site of apZH_gYSA_MMCas9 vector (Mikami et al. 2015). This construct was transformed into scutellum-derived NB calli via *Agrobacterium*-mediated transformation (Fukuoka et al. 2000). The mutation sites in the obtained T_1_ and T_2_ generation transformants were identified by Sanger sequencing. The *osdpd1dpd1-like* double mutant used in this study is a null segregant of the CRISPR-Cas9 transgene. The primer pairs used for Sanger sequencing were: Os03g0301400 SeqF1 (5’-CCTTGCTTGCAACCATTGAC-3’), Os03g0301400 SeqR1 (5’-AGTCACCTGCTTTGCTCCCA-3’), Os04g0623400 SeqF1 (5’-GCACTCTGTGATCTTTCTGG-3’), and Os04g0623400 SeqR1 (5’-CAGCAAATTCCAAGAACTACG-3’).

### PCR analysis

For genotyping analysis of GE lines, genomic DNA was extracted from fresh leaves of each line as described previously (Kamau et al. 2015). PCR was performed with a standard PCR setting (98°C for 30 sec/57°C for 30 sec/68°C for 30 sec, 35 cycles). To confirm the genotype of GE lines, degenerated cleaved amplified polymorphic sequence (dCAPS) and cleaved amplified polymorphic sequence (CAPS) primers were designed for *OsDPD1* and *OsDPD1-like*, respectively. Primers used for *OsDPD1* were OsDPD1 forward (5’-TTGAGTCGTCAAATGGCTGATAAACCAGAT-3’) and OsDPD1 reverse (5’-CACTTGACAAAAGAACGTACC-3’). Primers used for *OSDPD1-like* were OsDPD1-like forward (5’-TGTCACTCAACATGATGCTGTG-3’) and OsDPD1-like reverse (5’-AGTGAGAGTACTTACCCTCTGC-3’). PCR fragments were digested using *Mbo*I and genotyped by agarose gel electrophoresis to distinguish each genotype (Supplementary Fig. S1).

For qPCR analysis to estimate organelle DNA amounts, total DNA was isolated from flag leaves and pollen grains as described previously (Kamau et al. 2015; Matsushima et al. 2011). For isolating total DNA from pollen, pollen grains were collected in a microtube then disrupted using a pestle. To quantify plastid DNAs, primers for qPCR were designed in *PsbA* as follows: qPCR_PsbA forward (5’-ACATCGGATGGTTCGGTGTT-3’) and qPCR_PsbA reverse (5’-GATCGCCGCAGAAGTAGGAA-3’). The reactions were performed using a kit (Thunderbird SYBR qPCR Mix; Toyobo Co. Ltd.) and Light Cycler 2.0 software (Roche Diagnostics Corp.) with 40 cycles of denaturation (95 °C for 5 s) and extension (60 °C for 30 s). Quantitative data were obtained from at least three biological replicates and were analyzed using Light Cycler version 4.0 software (Roche Diagnostics Corp.). To normalize qPCR data from orgDNA over nuclear DNA, the *OsACTIN1* gene was used as a control of nuclear DNA with the following primers: qPCR_ACTIN1 forward (5’-GGATATGCTCTCCCCCATGC-3’) and qPCR_ACTIN1 reverse (5’-TCCCTCACAATTTCCCGCTC-3’).

For qRT-PCR to examine gene expression, total RNA was isolated using an ReliaPrep™ RNA Miniprep Systems (Promega), followed by reverse transcriptase and PCR reactions with a ReverTra Ace qPCR RT Kit (Toyobo Co. Ltd.) in accordance with the manufacturer’s instructions. We used the *OsACTIN2* gene as an internal control with the primers: qRT_OsACTIN2 forward (5’-TTCCAACAGATGTGGATCTCA-3’) and qRT_OsACTIN2 reverse (5’-GGACACCAACAATCCCAAAC-3’). The following primers were used to amplify the transcripts corresponding to each gene: qRT_OsSGR forward (5’-CGCTACTACATCTTCCGCAA-3’) and qRT_OsSGR reverse (5’-TGGAGTGGAAGTAGACCCACA-3’) for *OsSGR*, qRT_OsDPD1 forward (5’-ATGGACGTAGCTTTGATGTACC-3’) and qRT_OsDPD1 reverse (5’-TCAGTTTTTCCCCATCAGAACC-3’) for *OsDPD1*, and qRT_OsDPD1-like forward (5’-ATGCGCATAGAGCAATGCGAG-3’) and qRT_OsDPD1-like reverse (5’-ACATGGAACGGGAGCTTCATG-3’) for *OsDPD1-like*.

### Cytological observation

Pollen grains stained with SYBR Green I (1:500 dilution) were observed directly using a confocal laser-scanning microscope (Stellaris 5, Leica) with an HC PL APO CS2 63×/1.20 WATER immersion objective lens. SYBR Green I was excited using an 488 nm optically pumped semiconductor laser. For the detection of SYBR Green I fluorescence, a HyD detector was used with the detection range set at 493–738 nm.

### RNA seq analysis

Total RNA was isolated from leaves grown in -P, +P, or control conditions as described in the section on hydroponic growth. RNA sequencing was conducted using a DNBSEQ-G400RS (MGI) (Genome-Lead, Japan) and DNBSEQ-G400RS High-throughput Sequencing Set FCL PE150 (MGI), including sequencing library preparation using a KAPA mRNA Capture Kit (KAPA) and MGIEasy RNA Directional Library Prep Set (MGI). Sequences were obtained as pair-end reads. At least five billion reads were obtained for each sample (*n =* 3). Adaptor sequences were removed using fastp. Mapping of the obtained sequences was performed using HISAT2.

The gene expression levels were detected using the DESeq2/R package after read count calculations with feature Counts (Rsubread/R package) with Oryza_sativa.IRGSP-1.0.51.gtf. Volcano plots were constructed with the dataset of genes obtained using our RNA seq experiment (Supplementary Tables S1, S3 and S6 for -P, and Supplementary Tables S2, S4 and S7 for + P). The volcano plot in Fig. 6A was constructed using the dataset in Supplementary Table S5. Scatter plots were constructed by extracting three sets of the genes, designated as PSR (Supplementary Tables S8 and S11), P-indicator (Supplementary Tables S9 and S12), and P1BS (Supplementary Tables S10 and S13) gene sets, as reported previously (Jeong et al. 2017; Takehisa and Sato 2019; Wu et al. 2013). All plots were constructed using ggplot2/R packages. The VennDiagram/R package was used for Venn diagram construction. For STRING functional enrichment analysis, STRING 11.5 version and Cytoscape 3.9.1 version were used.

## Results

### Generation of a genome-edited line with both *DPD1* genes inactivated

Because AtDPD1 has been identified by forward genetics in *Arabidopsis*, we initially reasoned that *DPD1* was likely present as single copy. However, our phylogenic analysis revealed that *Arabidopsis* is an exception and most angiosperms appeared to possess two or more *DPD1* homologues (Chung et al. 2023; Takami et al. 2018). In rice, there are two *DPD1* homologues, annotated as *OsDPD1* (*Os03g0301400*) and *OsDPD1-like* (*Os04g0623400*), which showed 33% and 33% amino-acid alignments with *AtDPD1*, respectively, and 41% with each other using CLUSTALW (https://www.genome.jp/tools-bin/clustalw) (Fig. 1A). In silico analysis using public databases (https://rapdb.dna.affrc.go.jp) revealed that both *OsDPD1* and *OsDPD1-like* transcripts are more abundant in flowering buds than in other tissues, and *OsDPD1-like* has much lower expression levels than *OsDPD1*. According to the TargetP program (https://services.healthtech.dtu.dk/services/TargetP-2.0/), OsDPD1 was predicted to be predominantly targeted to chloroplasts, although showing some probability for mitochondrial targeting as well. On the other hand, *OsDPD1-like* has two gene models where alternative splicing gave rise to two transcripts generating a truncated version of OsDPD1-like proteins at the C-terminus (Fig. 1A). Both versions of OsDPD1-like were predicted to be targeted to mitochondria.

To investigate the effect of DPD1 nuclease, we attempted to knockout both genes using the CRISPR-CAS9 system, as described in Methods. The original vector for rice transformation (pZH_OsU6gRNA_MMCas9) contained an optimized *Cas9* and the guide RNA (gRNA) sequences, driven by 2 × *35 S* promoter and *OsU6* promoter, respectively. A 20-bp gRNA sequence (Fig. 1A), whose target site is located in the third exon of *OsDPD1* (one-base mismatch) and *OsDPD1-like* (complete match), was designed and inserted into our construct. After Agrobacterium-mediated transformation of rice calli derived from wild-type NB followed by hygromycin-resistance selection, we obtained six transgenic lines in the T1 generation. Among these lines, Sanger sequencing of the regions corresponding to both *OsDPD1* and *OsDPD1-like* revealed that one line contained chimeric mutations in both genes. This line was termed the genome-edited (GE) line and further characterized in this study.

### Verification of the mutations in *OsDPD1*and *OsDPD1-like* in the GE line

We verified the mutations in *OsDPD1* and *OsDPD1-like* in the GE line by Sanger sequence, in the subsequent T2 and T3 generations. For *OsDPD1*, a single nucleotide (T) was shown to be inserted within the target sequence, which resulted in a nonsense mutation generating a stop codon five amino acids after the insertion point (SVGRS) (Fig. 1B). For *OsDPD1-like*, unlike a canonical in/del or nucleotide substitution, we found that the nucleotide sequence disruption at the target site appeared to result from the deletion of 44 bp and the concomitant insertion of 43 bp of unknown origin (Fig. 1C). This modification created a missense mutation of 13 amino-acid residues (HVTQHGVEQRSNS) followed by a stop codon. Consequently, OsDPD1 and OsDPD1-like were mutated in the GE line by disruption of the C-terminal portion of each protein. Truncation at the C-terminal part of OsDPD1 and OsDPD1-like is likely to cause a loss-of-function mutation, as we determined for a similar *dpd1* allele (*dpd1-6*) in *Arabidopsis* created by T-DNA insertion in the third exon. We therefore considered that the GE line is a knockout mutant of organellar DNA degradation function mediated by DPD1 homologues.

We next confirmed homozygosity for *OsDPD1* and *OsDPD1-like* by generating CAPS markers that distinguished the mutant and wild-type alleles between NB and GE (Supplementary Fig. S1, primers are indicated in the Methods). We selected individuals that were homozygous for both mutations in the T2 generation. These lines no longer harbored the transgene insertion. We harvested seeds from these T2 individuals, and the resulting seeds from T3 and T4 generations were subjected to further phenotypic analyses.

### Gene expression and cpDNA amount during dark-induced and natural senescence

**Fig. 2:**
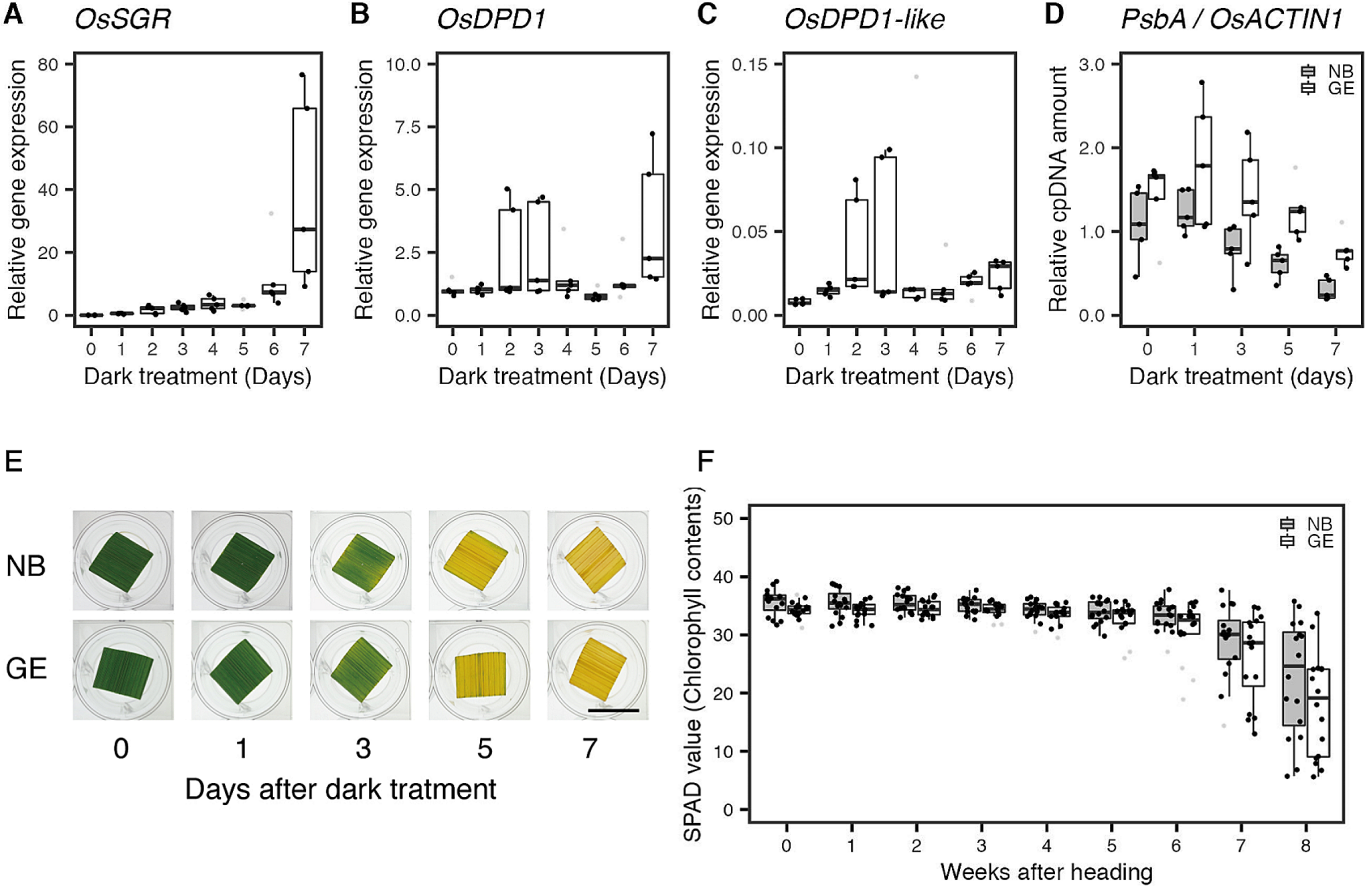
Gene expression and cpDNA amounts during dark-induced senescence, and chlorophyll content in natural senescence. Transcript levels of *OsSGR* (**A**), *OsDPD1* (**B**), and *OsDPD1-like* (**C**) in NB senescing flag leaves, estimated by qRT-PCR.*OsACTIN* was used as an internal control in these analyses (*n* = 5). (**D**) Relative amounts of cpDNAs in NB and GE senescing flag leaves, measured by qPCR (gray and white boxplots represent NB and GE, respectively, *n* = 5). (**E**) A representative image of leaves from NB (top) and GE (bottom), subjected to dark-induced leaf senescence as indicated. Scale bar = 1 cm. (**F**) Measurement of chlorophyll content in natural flag-leaf senescence during summer (gray and white boxplots represent NB and GE, respectively, *n* = 16). The trend in relative gene expression level was analyzed using the Jonckheere-Terpstra test with a PMCMRplus/R package. The trend in OsSGR gene expression showed a significant increase (*p* = 2.91e-10). The trend in OsDPD1 gene expression showed a significant increase (*p* = 0.0223). The trend in OsDPD1-like gene expression showed a significant increase (*p* = 0.00756). The trend in relative cpDNA amount in NB showed a significant decrease (*p* = 1.96e-5). The trend in relative cpDNA amounts in GE showed a significant decrease (*p* = 9.98e-4). The trend in chlorophyll content in NB showed a significant decrease (*p* = 9.55e-14). The trend in chlorophyll content in GE showed a significant decrease (*p* = 1.25e-13). For boxplots, the lower and upper whiskers indicate minimum and maximum values, respectively. The bottom, center, and top lines of the box indicate the lower quartile, median, and upper quartile, respectively

In our previous study, expression of DPD1 exonuclease was shown to be upregulated during leaf senescence and contributed to abundant cpDNA degradation in *Arabidopsis* and *Populus* (Takami et al. 2018). To investigate the same effects of *OsDPD1* and *OsDPD1-like* on leaf senescence in rice, we first measured the gene expression levels of *OsDPD1* and *OsDPD1-like* in NB by qRT-PCR analysis after dark treatment (0,1,2,3,4,5,6, and 7 days). As a positive control, *OsSGR* was used and showed the typical induction pattern as reported previously (Yamatani et al. 2013), confirming the onset of dark-induced leaf senescence (Fig. 2A). Our qRT-PCR results showed that *OsDPD1* and *OsDPD1-like* transcript levels appeared to increase only marginally (Fig. 2B and C).We also measured the amount of cpDNAs in both NB and GE after dark treatments (0, 1, 3, 5 and 7 days). Results revealed that no significant difference was observed between NB and GE, although cpDNAs were shown to be degraded in both lines (Fig. 2D). It was thus likely that other mechanisms than DPD1, such as the degradation of chloroplasts by autophagy, may contribute to orgDNA degradation (see Discussion). Consistent with these results in cpDNA amounts, we observed no significant difference in stay-green phenotype in the GE line, for both dark-induced leaf senescence (Fig. 2E) and natural leaf senescence in the flag leaves (Fig. 2F). Based on these observations, it is likely that cpDNA degradation has little impact on rice leaf senescence.

### Gene expression and cpDNA amount in pollen

**Fig. 3:**
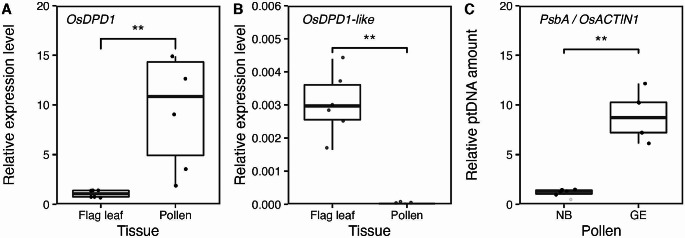
Gene expression and cpDNA amounts in pollen. (**A**) Comparison of *OsDPD1*transcript levels between flag leaves and pollen grains (*n* = 6). (**B**) Comparison of *OsDPD1-like* transcript levels between flag leaves and pollen grains (*n* = 6). *OsACTIN1* was used as an internal control in these analyses. (**C**) Relative amounts of cpDNAs in NB and GE pollen grains (*n* = 6), measured by qPCR. Statistical analysis of data was performed using the Wilcoxon-Mann-Whitney test with the ggsignif/R package, with significance levels at *p* < 0.01(**), and *p* < 0.001(***). For boxplots, the lower and upper whiskers indicate the minimum and maximum values, respectively. The bottom, center, and top lines of the box indicate the lower quartile, median, and upper quartile, respectively

**Fig. 4:**
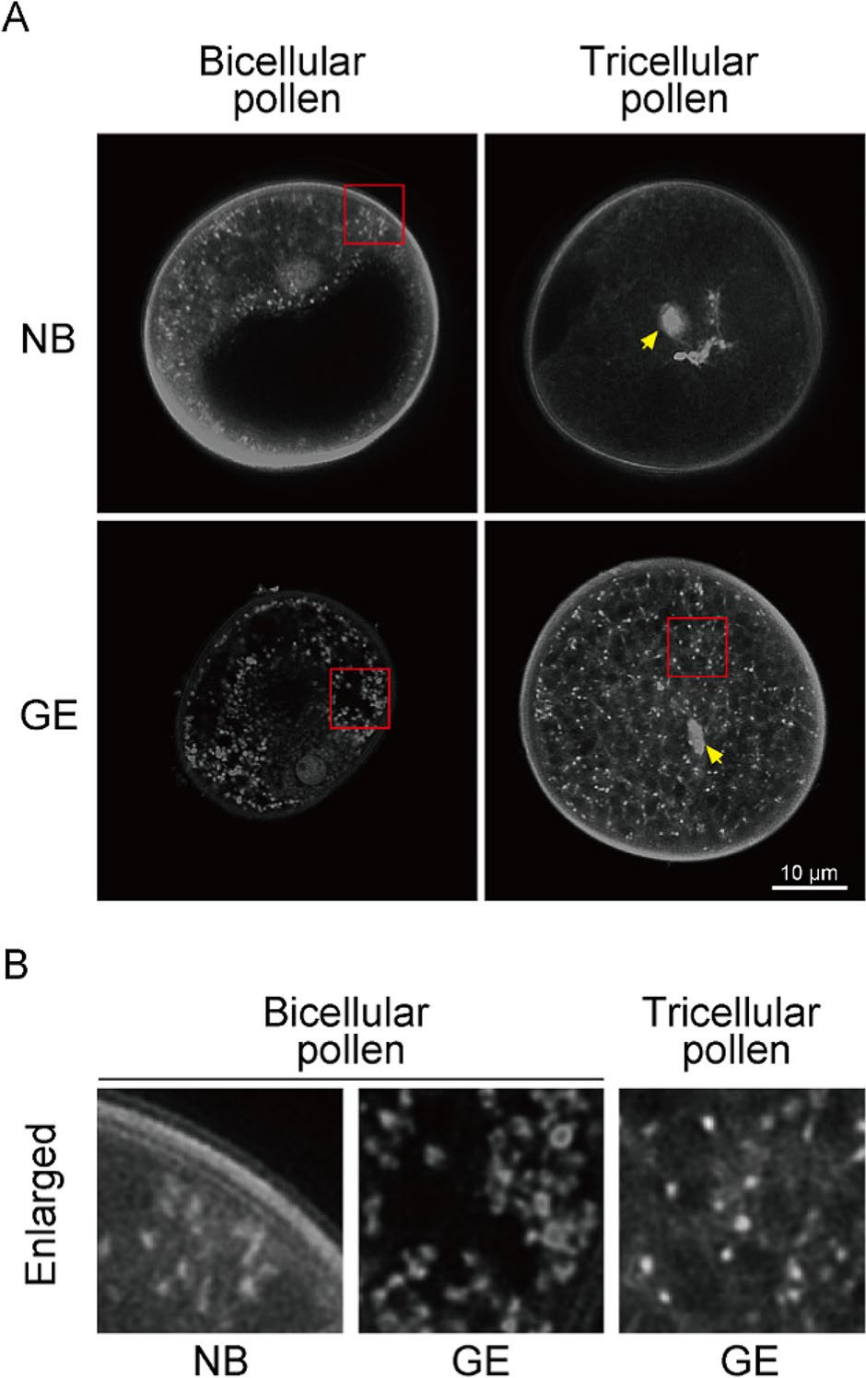
Confocal-microscopic observations of bicellular and tricellular pollen grains from NB and GE. (**A**) Representative images of bicellular (left) and tricellular (right) pollen grains from NB (top) and GE (bottom) stained with SYBR-Green and detected using confocal laser scanning microscopy. Note that small dot-like signals corresponding to orgDNAs were detected in bicellular pollen from both NB and GE, whereas these signals in tricellular pollen were detected only in GE. Yellow arrows show the signals corresponding to vegetative nuclei. Observation of pollen grains in NB and GE plants was performed with at least three biological replicates and the representative images is shown. (**B**) Closeup view of bicellular (left, middle) and tricellular (right) pollen, enlarged from the areas indicated red in (**A**)

Next, we measured the expression levels of *OsDPD1* and *OsDPD1-like* using qRT-PCR in pollen grains, because DPD1 expression was shown to be enhanced in *Arabidopsis* and tobacco to accelerate orgDNA degradation (Chung et al. 2023; Takami et al. 2018). To compare the expression in pollen, we also measured *OsDPD1* and *OsDPD1-like* transcripts in flag leaves. The results showed that *OsDPD1* was highly induced in pollen grains (Fig. 3A). In contrast, almost no *OsDPD1-like* expression was observed in mature pollen grains. In general, the expression of *OsDPD1-like* was low in most tissues, and it was barely expressed in flag leaves (Fig. 3B). We also measured the amount of cpDNA in NB and GE pollen grains using qPCR and cytological observations. Consistent with the elevated expression of *OsDPD1*, cpDNAs were retained only in GE but not in NB pollen grains (Fig. 3C). Furthermore, confocal microscopic observations of DNAs in mature pollen using SYBR Green staining showed that bicellular pollen had fluorescent signals corresponding to orgDNAs as nucleoids in both NB and GE lines, whereas tricellular pollen retained these spots only in the GE line (Fig. 4). Fluorescent spots were numerous and appeared more than the number of plastids (Matsushima et al. 2008), likely representing nucleoids in both plastids and mitochondria; we detected two types of signals, one represented by large, bright spots and the other by small, faint spots, which might represent cpDNAs and mtDNAs, respectively (Fig. 4B). Together, these results indicated that orgDNAs are degraded by DPD1 exonuclease in rice, similar to in *Arabidopsis*.

### Comparative analysis of transcriptome profiles

Our previous study on *Arabidopsis dpd1* indicated that orgDNA degradation contributes to efficient phosphate recycling and seed setting in phosphorus-depleted growing conditions (Takami et al. 2018). This ‘phosphate reservoir’ function of DPD1 was implied from the *Arabidopsis dpd1* mutant, based on (i) the diminished expression of the set of genes responding to phosphate starvation (see below) and (ii) lower seed production in -P conditions. To examine if this is also the case in rice, we grew NB and GE plants in hydroponic cultures similar to the previous study. We set up phosphate sufficient (+ P) and depleted (-P) conditions. NB and GE seedlings grown in normal 1/10 MS media were subjected to either -P (deprivation) or + P (2 mM Pi) conditions, and subsequently RNA seq analysis was performed on 10-day-old seedlings exposed to these treatments. These expression data were compared with control RNA seq data from untreated NB and GE plants.

**Fig. 5:**
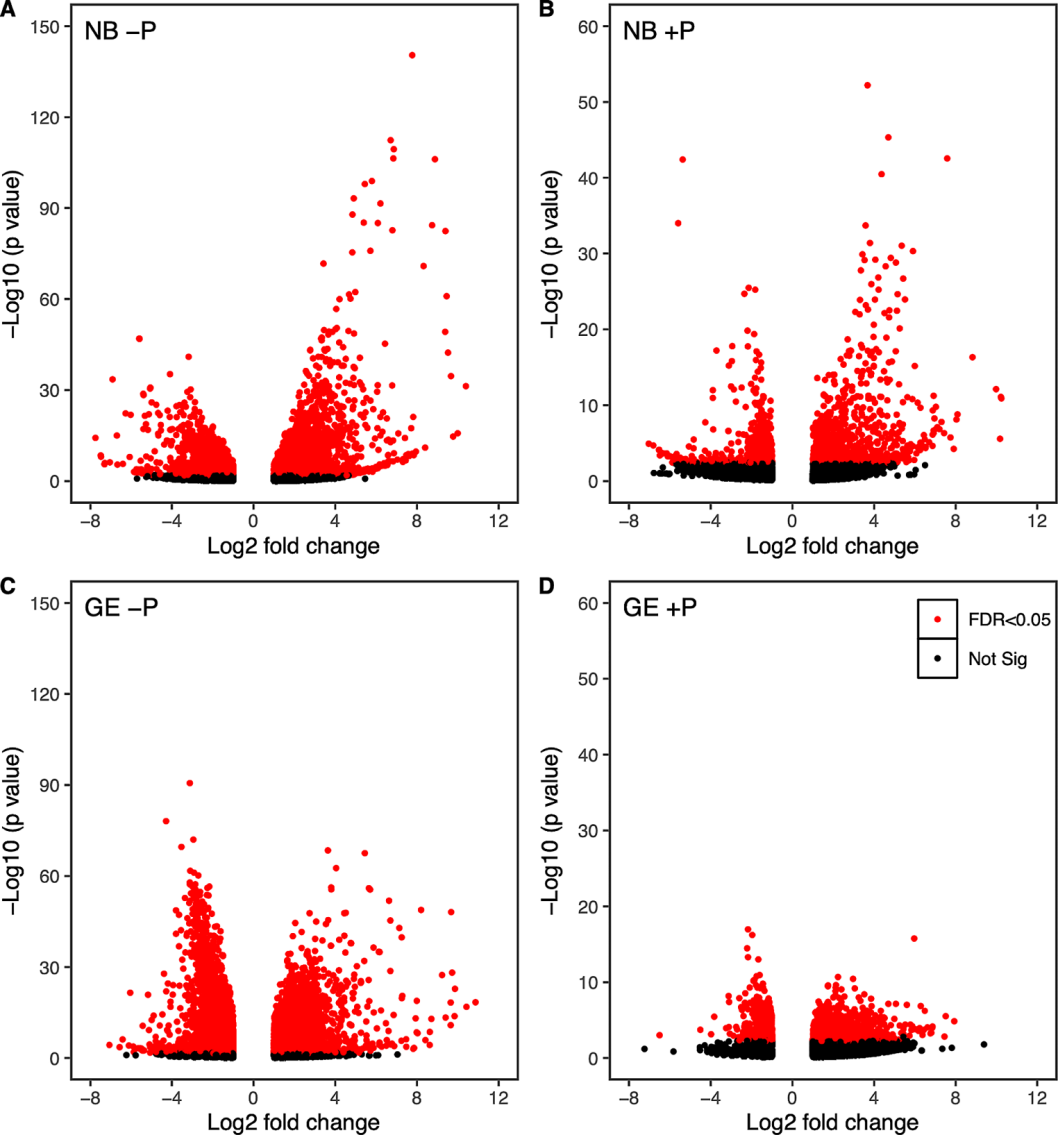
Volcano plot analysis of transcriptome responding to -P and + P (comparison of transcriptome between normal and phosphate-altered conditions in NB and GE). (**A**) Volcano plots showing significantly differentially expressed genes in NB in -P conditions. (**B**) Volcano plots showing significantly differentially expressed genes in NB in + P conditions. (**C**) Volcano plots showing significantly differentially expressed genes in GE in P conditions. (**D**) Volcano plots showing significantly differentially expressed genes in GE in + P conditions. Volcano plots were constructed using the ggplot2/R package with the data set of all DEGs. Each data point represents a single gene. Threshold levels for DEGs were log2-fold change > 1 and adjusted *p*-value < 0.05

First, we characterized differentially expressed genes (DEGs) with -P or + P shifts, where threshold levels for DEGs are set as log2-fold change > 1 and an adjusted *p*-value < 0.05. For ‑P conditions, 2653 genes were upregulated and 2354 genes were downregulated in NB, whereas 4632 genes were upregulated and 3408 genes were downregulated in GE (Fig. 5A and C). For + P conditions, 813 genes were upregulated and 575 genes were downregulated in + P conditions in NB, whereas 1449 genes were upregulated and 660 genes were downregulated in the GE line (Fig. 5B and D). In contrast to our previous study in *Arabidopsis* in which DEGs in -P conditions were dramatically decreased in the *dpd1* mutant, we found that DEGs in -P conditions increased in the GE line rather than NB. Similarly to -P, DEGs responding to + P appeared to increase considerably in the GE line (Fig. 5B and D), suggesting that rice plants responded to phosphate supply differently compared with *Arabidopsis*.

**Fig. 6:**
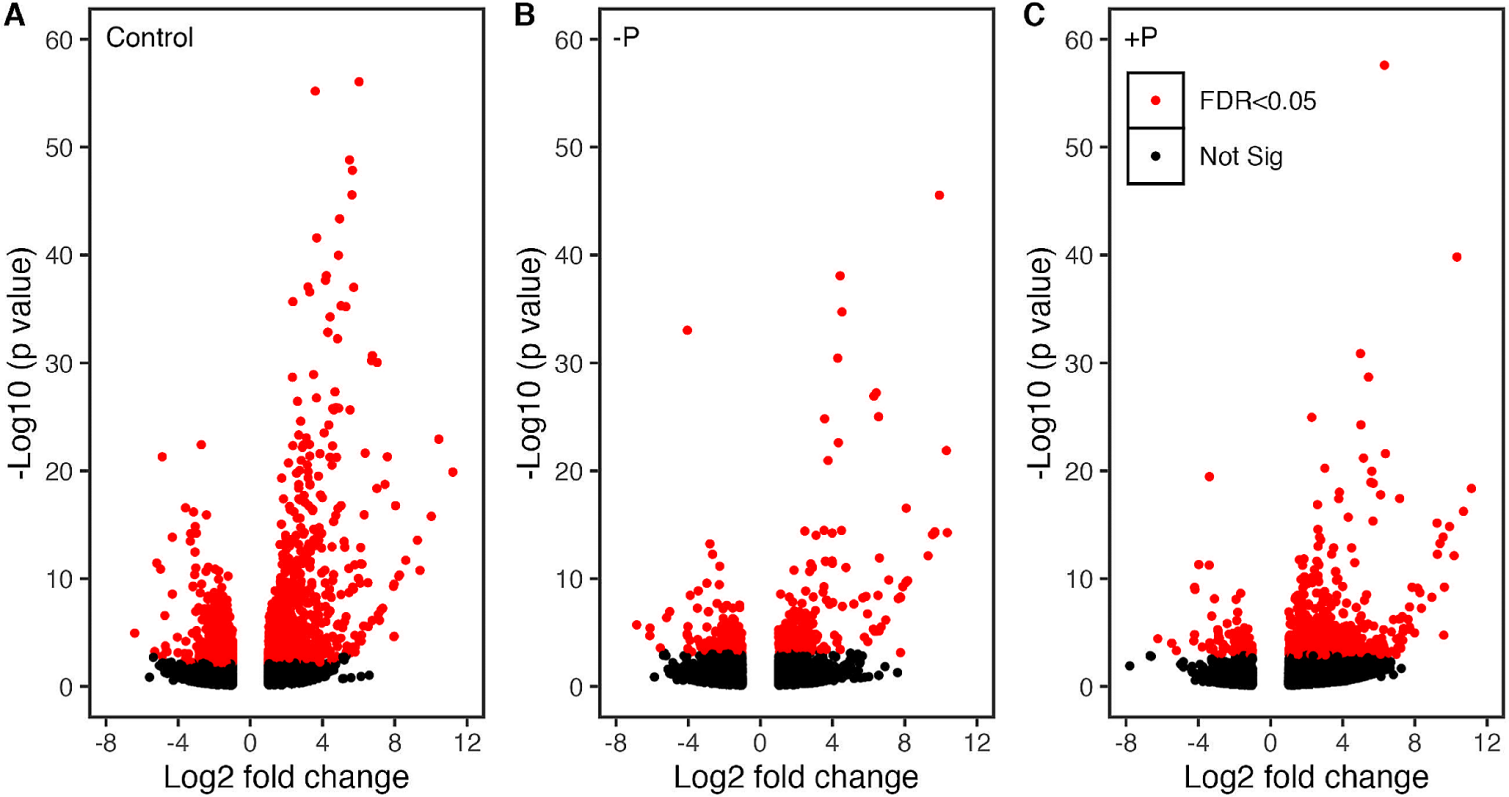
Comparison of transcriptomes between NB and GE from leaf tissues grown in normal, -P, and + P condition. (**A**) Volcano plot showing differentially expressed genes between NB and GE in normal (1/10MS medium) conditions. (**B**) Volcano plot showing differentially expressed genes between NB and GE in -P conditions. (**C**) Volcano plot showing differentially expressed genes between NB and GE in + P conditions. Volcano plots were constructed using the ggplot2/R package with the data set of all DEGs. Each data point represents a single gene. Threshold levels for DEGs were log2-fold change > 1 and adjusted *p*-value < 0.05

Because there were significant numbers of genes up- or down-regulated in the GE line, we next compared the overall transcriptome between NB and GE, rather than a comparison between -P or + P and normal growth conditions in each line. Comparison of DEGs using volcano plots between NB and GE in control conditions showed that 995 genes were upregulated and 1418 genes were downregulated (Fig. 6A). In contrast, comparison of DEGs between NB and GE in ‑P and + P conditions indicated that the number of the DEGs was apparently more in control conditions; for -P conditions, 220 genes were upregulated and 158 genes were downregulated (Fig. 6B). On the other hand, for + P conditions, 615 genes were upregulated and 82 genes were downregulated (Fig. 6C). Gene ontology (GO) analysis with control condition showed that GO biological category such as response to stress (GO:0006950), regulation of jasmonic acid-mediated signaling pathway (GO:2,000,022), response to ethylene (GO:000972), and response to abiotic stimulus (GO:0009628) were enriched in the GE line (S18). These results suggested that orgDNA degradation may have more diverse effects in plant growth in addition to P starvation.

### Response of NB and GE to -P and + P conditions

**Fig. 7:**
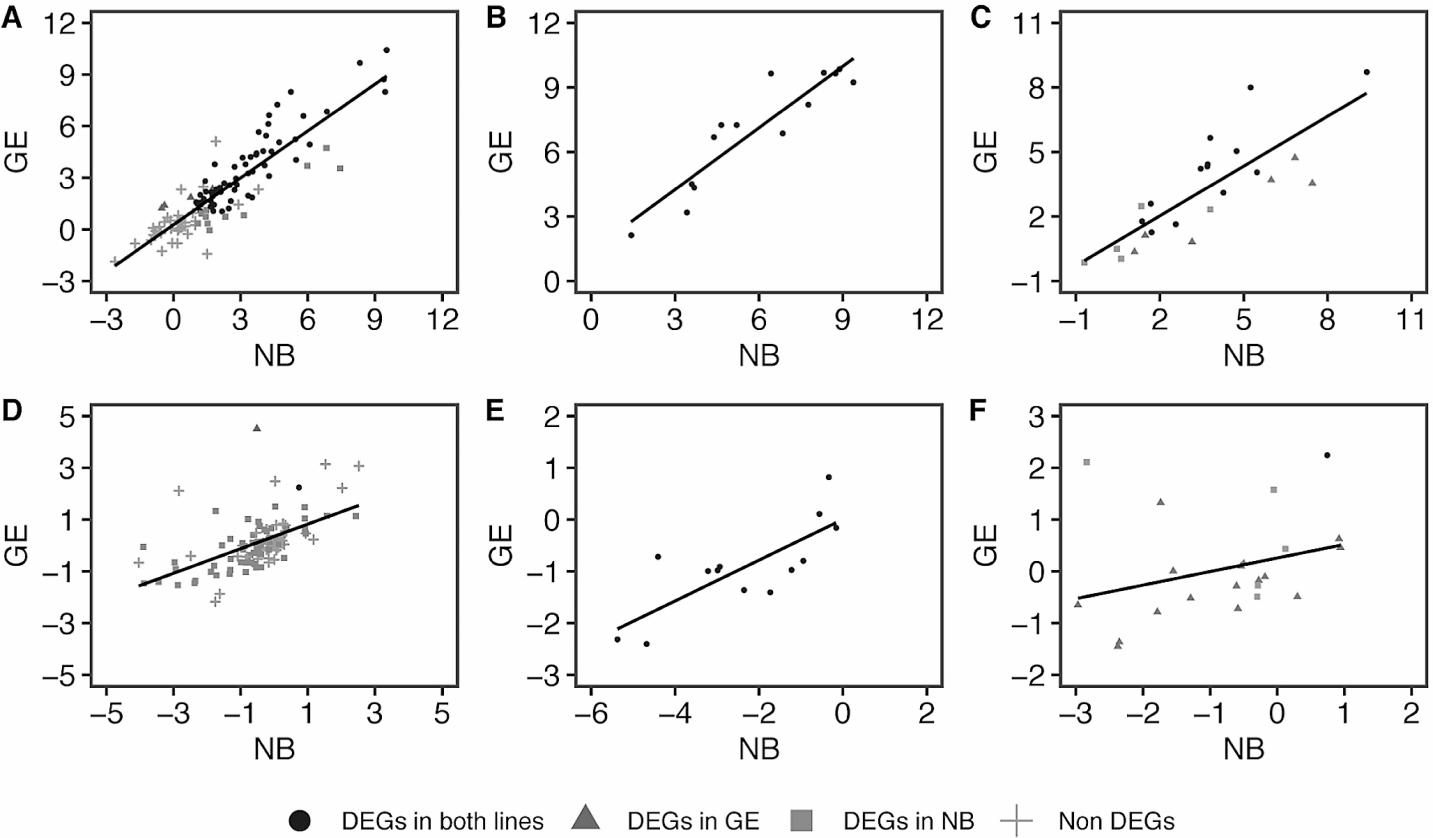
Scatter plot analysis of gene expression between NB and GE in three gene sets known to respond to phosphate starvation. (**A**) Scatter plot of the genes in the PSR gene set in -P conditions. (**B**) Scatter plot of the genes in the P-indicator gene set in -P conditions. (**C**) Scatter plot of the genes in the P1BS gene set in -P conditions. (**D**) Scatter plot of the genes in the PSR gene set in + P conditions. (**E**) Scatter plot of the genes in the P-indicator gene set in + P conditions. (**F**) Scatter plot of the genes in the P1BS gene set in + P conditions. We used different symbols for DEGs in this analysis. Filled circle, filled triangle, filled square and plus show DEGs in both lines, DEGs in GE, DEGs in NB and non DEGs, respectively. Each data point represents a single gene

To further investigate the possible response to P supply in the GE line, we specifically analyzed three sets of genes categorized as related to the response to phosphate depletion, namely the phosphate starvation response (PSR), P-indicator, and PHR1 binding sequences (P1BS) genes (Jeong et al. 2017; Takehisa and Sato 2019; Wu et al. 2013). Genes categorized in these gene sets were extracted from our RNA seq dataset, and their expression levels were compared between NB and GE using scatter plots (Fig. 7). In case of -P, the expression patterns of the PSR, P- indicator and P1BS genes showed a positive correlation between NB and GE (Fig. 7A and C and Supplementary Table S21). In both NB and GE genotypes, the genes encoding phosphate transporters (*OsPT1*, *OsPT4*, *OsPT10*, *OsPT8, OsPHO1;2*, and *OsPHO1;3*), purple acid phosphatase (*OsPAP3b*, *OsPAP23*, *OsPAP10a*, *OsPAP1d*, *OsPAP3c*, and *OSPAP21b*), SPX domain-containing proteins (*OsSPX1*, *OsSPX2*, *OsSPX3*, and *OsSPX5*), glycosyl transferase (*OsSQD2*), acid phosphatase (*OsACP1*), and RNases (*OsRNS3* and *OsRNS6*) were upregulated (Fig. 7A). Among the P-indicator genes, 14 genes were differentially expressed in both NB and GE plants and also upregulated (Fig. 7B). Of 27 P1BS genes, 14 genes were differentially expressed in both NB and GE plants, 6 genes were differentially expressed only in NB plants, and 7 genes showed no differential expression (Fig. 7C).These results indicated that the overall response to PSR appeared to be similar between NB and GE plants.

Next, we examined the PSR, P-indicator, and P1BS genes in + P conditions. In contrast to the case in -P conditions, a positive correlation between NB and GE plants was no longer apparent in PSR and P1BS gene sets (Fig. 7D and F and Supplementary Table S21). Among 115 PSR genes, only a limited number of genes were upregulated in NB plants, including phosphate transporter (*OsPT1*) and transcription factor (*OsbHLH035*). Similarly, a phosphate transporter gene (*OsPT2*) was upregulated in GE plants (Fig. 7D). These results appeared to support the possibility that orgDNA degradation contributes to altered P supply in rice, but the response to PSR is not as prominent as those observed in *Arabidopsis*.

### Impact of orgDNA degradation assessed by agronomic traits in NB and GE plants

**Fig. 8:**
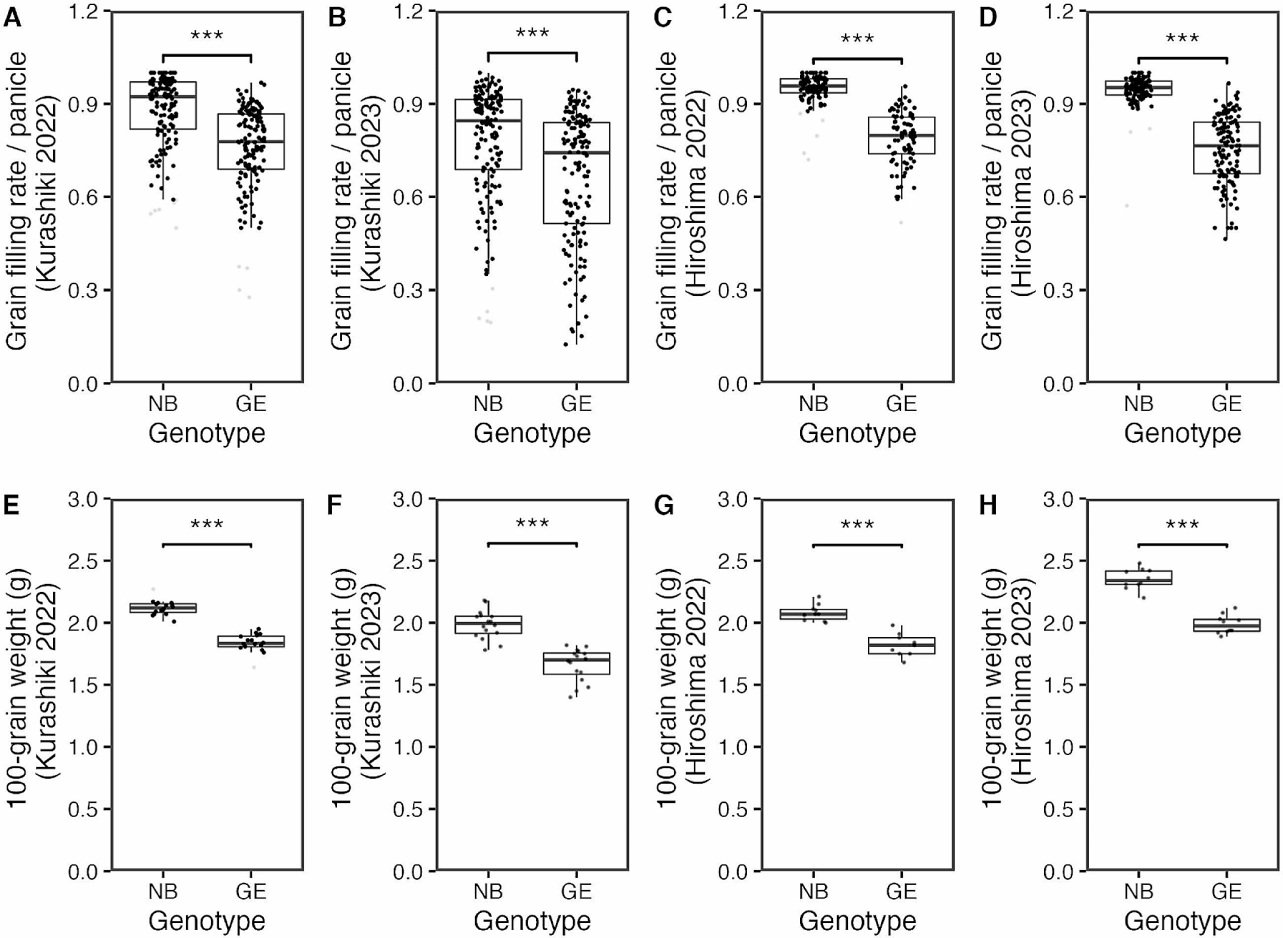
Grain filling rate and 100-grain weight of NB and GE plants grown in greenhouse conditions. Grain filling rate per panicle, measured by NB and GE plants grown in a greenhouse at Kurashiki (*n* = 141 and 147 panicles from 16 NB and 16 GE plants, respectively) in 2022 (**A**) and (*n* = 152 and 150 panicles from 16 NB and 16 GE plants, respectively) 2023 (**B**), or at Hiroshima(*n* = 90 and 82 panicles from 10 NB and 10 GE plants, respectively) in 2022 (**C**) and (*n* = 103 and 126 panicles from 10 NB and 10 GE plants, respectively)2023 (**D**) are shown. The weight of 100 grains, measured from NB and GE plants grown in a greenhouse at Kurashiki (*n* = 16 plants) in 2022 (**E**) and 2023 (**F**), and at Hiroshima (*n* = 10 plants) in 2022 (**G**) and 2023 (**H**) are shown. For boxplots, the lower and upper whiskers indicate the minimum and maximum values, respectively. The bottom, center, and top lines of the box indicate the lower quartile, median, and upper quartile, respectively. Statistical analysis of the data was performed using the Wilcoxon–Mann-–Whitney test with the ggsignif/R package, with significance at *p* < 0.001(***)

To examine the effect of orgDNA degradation on plant growth, we cultivated rice plants (NB and GE) in greenhouse conditions at two different locations (Kurashiki and Hiroshima) during the summer season in 2022 and 2023. In this trial, we attempted to assess how the lack of orgDNA degradation in the GE line influenced yield components, particularly those in the reproductive stage. Several agronomic traits, including grain filling, grain weight, panicle length, as well as plant height and dry weight, were compared between NB and GE plants. The results showed that most of the traits related to grain yield were significantly reduced in the GE line; grain filling rate per panicle (Fig. 8A and D), panicle length per panicle (Supplementary Fig. S3E-S3H), and 100-grain weight (Fig. 8E and H) all decreased in all locations and seasons. In contrast, plant height and dry weight per plant did not show any significant difference between NB and GE plants in 2022 (Supplementary Fig. S3A and S3I), although these were significantly decreased in GE plants in 2023 (Supplementary Fig.S3B and S3J). Together, we concluded that GE plants had lower seed setting, which was likely attributed to the increased number of sterile spikelets, showing reduced fitness in our growing conditions. Because only one GE line has been created in this study, however, we cannot completely rule out the possibility that the defective phenotypes were due to other mutations.

## Discussion

OrgDNA degradation and the responsible nucleases have been scarcely studied in rice. For example, cytological observations of mitochondria in roots demonstrated various DNA amounts in each mitochondrion (Takanashi et al. 2006). Egg cells appeared to contain much more mtDNAs than leaf cells (Takanashi et al. 2010). During the senescence of rice coleoptiles, ptDNAs were shown to be degraded as a characteristic event prior to organelle degradation (Inada et al. 1998). However, orgDNA degradation in rice pollen has not been examined in detail. Taking advantage of genome-editing technology using the CRISPR-CAS9 system, we successfully obtained a rice mutant defective in DPD1 nuclease activity in this study, and we examined the physiological role of orgDNA degradation in rice.

### Predominant role of DPD1 in orgDNA degradation in rice pollen

Our data using pollen tissues from NB and GE plants demonstrated that orgDNAs are degraded during rice pollen maturation by DPD1 (Fig. 4). Based on the expression levels of *OsDPD1* and *OsDPD1-like* in pollen grains (Fig. 3A and B), we consider *OsDPD1* as the predominant exonuclease responsible for orgDNA degradation, not only in pollen but also in the other tissues. Apparently, *OsDPD1* is highly upregulated in pollen, whereas almost no *OsDPD1-like* transcripts were detected in pollen. In general, the expression level of *OsDPD1-like* appeared to be extremely low. Although further analysis is needed, our data suggest that OsDPD1 degrades both ptDNA and mtDNA, similarly to AtDPD1. In rice, ptDNA is maternally inherited, likely due to the exclusion of plastids and the degradation of orgDNAs in sperm cells. However, whether there is leakage of paternal ptDNAs at low frequency (~ 10^− 5^), as found in *Arabidopsis* and tobacco (Azhagiri and Maliga 2007; Ruf et al. 2007; Svab and Maliga 2007), remains unclear. In tobacco, a recent study demonstrated that DPD1 acts in securing the maternal inheritance of ptDNA, because a lack of DPD1 resulted in a > 100-fold increase of paternal leakage. The GE plant created in this study is suited for such analysis in the future, although selectable markers for paternal plastid inheritance may be needed in rice.

### Effects of DPD1 in leaf senescence

Leaf senescence is the final stage of leaf development and the last step in the life cycle for many plant species, which play a role in the efficient recycling and redistribution of nutrients. For example, nitrogen is the major source for such redistribution, and most nitrogen is stored as macromolecules such as proteins and nucleic acids in chloroplasts. Given these facts, chloroplasts are considered as the major target for degradation processes during leaf senescence (Makino and Osmond 1991). Previously, Inada et al. cytologically characterized leaf senescence in rice coleoptiles, in which sequential degradation processes of cellular organelles were carefully observed (Inada et al. 1998). They observed cpDNA degradation as one of the earlier events characteristic to the senescing leaves, corroborating that cpDNA degradation took place in rice (NB). Supporting their observations, our qPCR data (Fig. 2D) also showed that cpDNA levels declined in leaves undergoing dark-induced leaf senescence.

In this study, we evaluated if DPD1 is responsible for this cpDNA degradation by analyzing NB and GE plants. In contrast to our previous observation in *Arabidopsis*, however, the involvement of DPD1 in cpDNA degradation was not clear; although a slight increase in cpDNA levels was detected in GE plants compared with NB, the difference was not significant. Concomitantly, we also observed only marginal increases in *OsDPD1* expression. One possibility to explain these observations is the presence of multiple pathways to induce leaf senescence in rice. Leaf senescence is regulated by age-dependent and environmental conditions, and internal cues such as phytohormonal signals, water usage, nutrient status, light quality and length, and climate change can influence the onset and intensity of leaf senescence (Woo et al. 2019). Cereal crops such as rice have been developed during breeding programs to maximize ‘harvest index’, representing the efficient relocation of nutrients to upper tissues (Izumi et al. 2015; Wada et al. 2015). Although cpDNA degradation is implemented by DPD1 exonuclease, its contribution may not be as significant in rice as in *Arabidopsis*. For example, autophagy is known to play an important role in chloroplast degradation (Izumi et al. 2015; Wada et al. 2015). Such a mechanism might act coordinately during leaf senescence in rice.

### Possible function of DPD1 in global plant growth in rice

We attempted to understand the role of orgDNA degradation in efficient phosphate redistribution. To examine this, the transcriptome was compared in NB and GE plants grown in different Pi conditions. In *Arabidopsis*, the *dpd1* mutant apparently showed diminished responses to phosphate starvation in -P conditions and its disturbed response was reinforced by extracting a set of genes known as PSR (Takami et al. 2018). In contrast to these findings in *Arabidopsis*, rice GE plants had increased DEGs in -P conditions (Fig. 5), suggesting that the lack of DPD1 has more profound effects in transcriptomes in rice, regardless of P supply conditions. Somewhat surprisingly, we consistently observed increased DEGs in not only -P but also + P conditions. Global effects in transcriptomes included various biological functions, such as response to temperature stimulus (GO:0009266), secondary metabolic process (GO:0019748), and response to biotic stimulus (GO:0009607), indicated by our functional enrichment analysis (Supplementary Fig.S2 and Supplementary Table S14-S20). Overall, these observations led us to consider that orgDNA degradation has more general impact in plant growth, by controlling orgDNA levels during plant development. Supporting these inferences is the lower seed setting (Fig. 8), reduced grain filling rate, and 100-grain weight in the GE line. We should be cautious to interpret these results, as we obtained only one GE line that may have additional mutations. The presence of off-target mutations was unlikely, because the 20-bp gRNA is highly specific to *OsDPD1* and *OsDPD1-like* with no potential off targets, based on the prediction by CRISPR direct (http://crispr.dbcls.jp). In addition, T3 and T4 individuals derived from different T2 lines showed similar phenotypes with no obvious segregation. Although further study is needed to conclude that low seed setting is associated with disturbed pollen orgDNA degradation or a more general function of DPD1 for fitness, our data confirm the versatile role of orgDNA degradation mediated by DPD1.

In conclusion, our analysis of DPD1 in the current study with rice demonstrated that tissue-specific degradation of orgDNA consistently takes place in pollen tissues, which is predominantly controlled by DPD1. In contrast, cpDNA degradation during senescence appeared to be controlled by multiple mechanisms, and the contribution of DPD1 may be different among species. OrgDNA degradation may contribute to efficient phosphate and other nutrient redistribution during plant growth, but its influence and impact may differ among species. In general, fine tuning of orgDNA levels is important, because the complete loss of cpDNAs in chloroplasts may eventually lead to cell death. Recent finding of DPD1 responding to biotic stress (Nair et al. 2023) implicates such a critical role.

### Electronic supplementary material

Below is the link to the electronic supplementary material.

Supplementary Material 1

Supplementary Material 2

Supplementary Material 3

## Data Availability

The original contributions presented in this study are included in the article and Supplemental materials. Further inquiries can be directed to the corresponding author. All RNA seq data obtained from this study have been deposited with links to BioProject accession number PRJDB17681 in the DDBJ BioProject database.
