# Supplementary figures and images for: Characterization of organelle DNA degradation mediated by DPD1 exonuclease in the rice genome-edited line
Source: Plant Mol Biol. 2024 Jun 10;114(3):71. doi: 10.1007/s11103-024-01452-x (PMC11164812; doi:10.1007/s11103-024-01452-x)

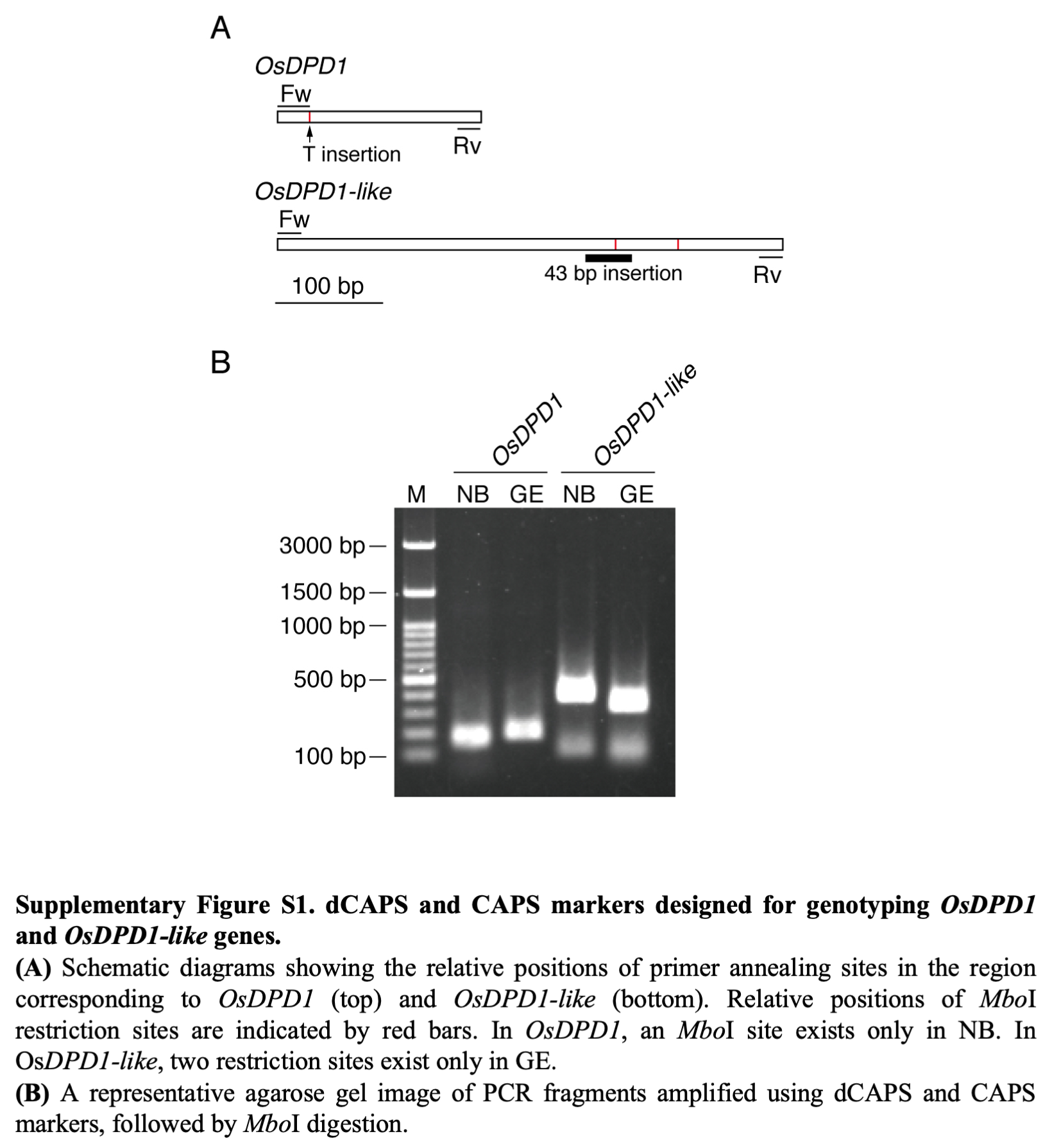

Supplement: Supplementary file 1 — Supplementary Material 1 [file 11103_2024_1452_MOESM1_ESM.tiff]

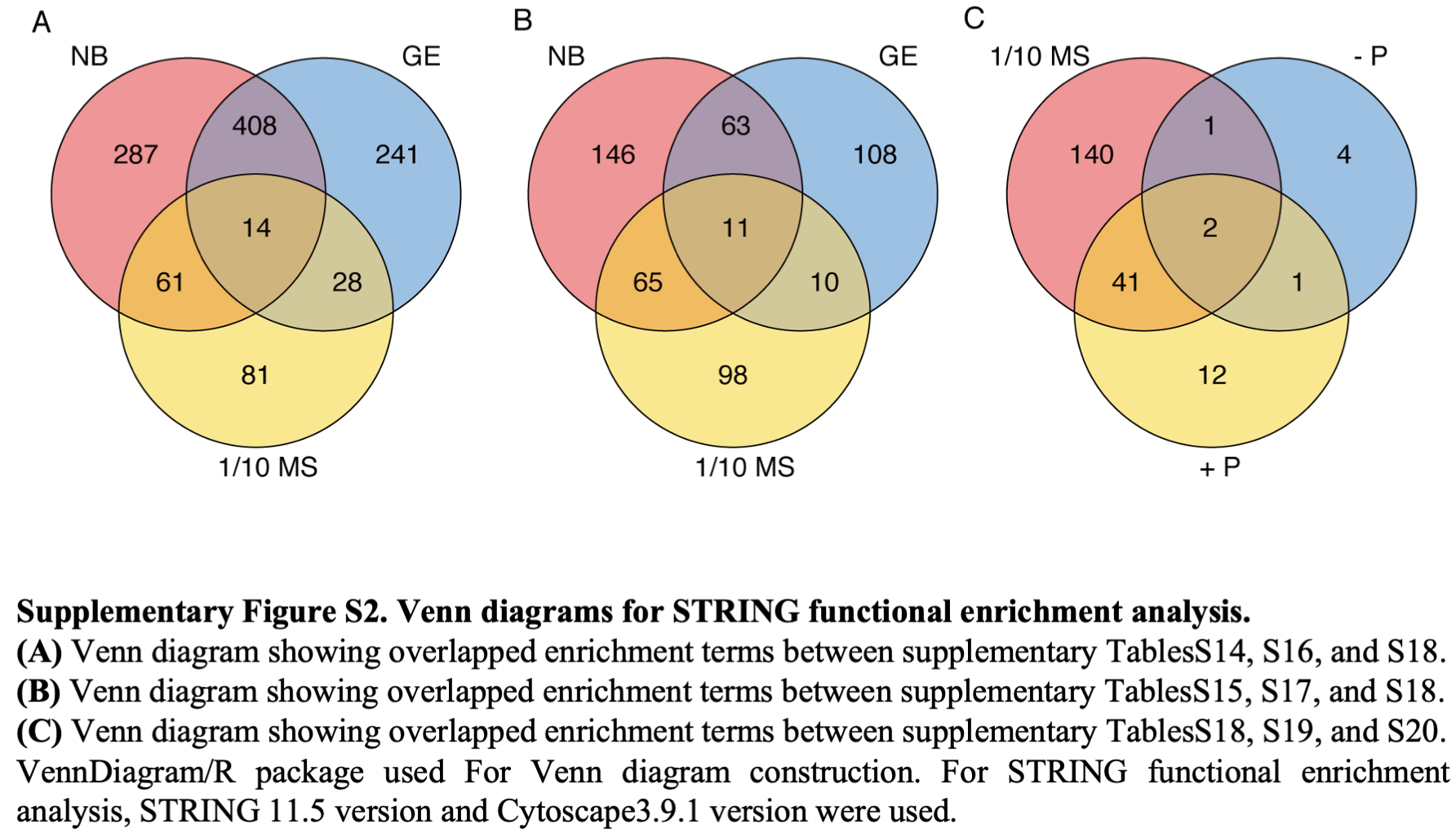

Supplement: Supplementary file 2 — Supplementary Material 2 [file 11103_2024_1452_MOESM2_ESM.tiff]

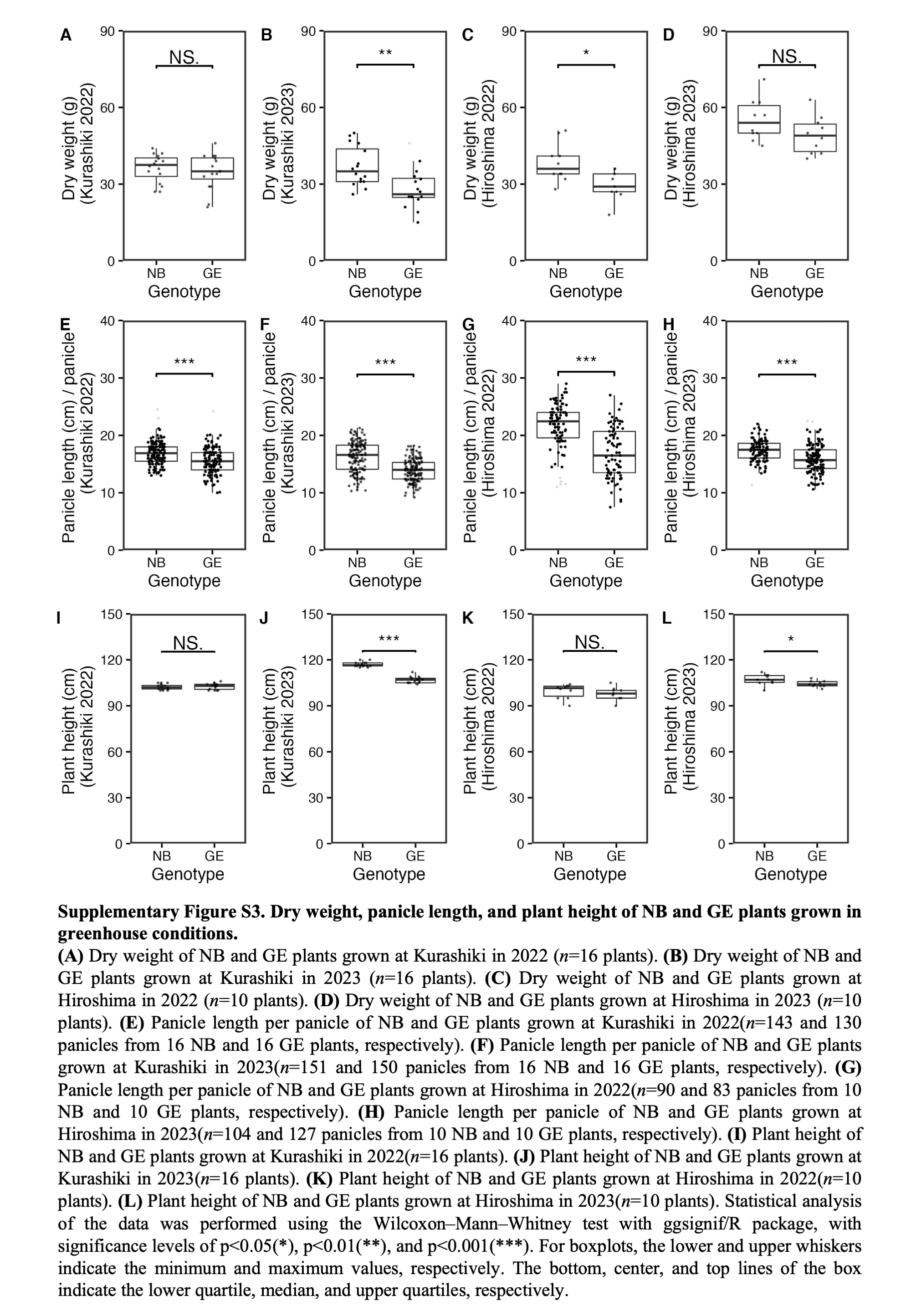

Supplement: Supplementary file 3 — Supplementary Material 3 [file 11103_2024_1452_MOESM3_ESM.tiff]
